# Potentially Bioactive Metabolites from Pineapple Waste Extracts and Their Antioxidant and α-Glucosidase Inhibitory Activities by ^1^H NMR

**DOI:** 10.3390/foods9020173

**Published:** 2020-02-11

**Authors:** Awanis Azizan, Ai Xin Lee, Nur Ashikin Abdul Hamid, Maulidiani Maulidiani, Ahmed Mediani, Siti Zulaikha Abdul Ghafar, Nur Khaleeda Zulaikha Zolkeflee, Faridah Abas

**Affiliations:** 1 Laboratory of Natural Products, Institute of Bioscience, Universiti Putra Malaysia, Serdang 43400, Selangor, Malaysia; awanis_azizan@yahoo.com (A.A.); shikinhamid89@yahoo.com (N.A.A.H.); khaleeda_zulaikha@yahoo.com (N.K.Z.Z.); 2 Department of Food Science, Faculty of Food Science and Technology, Universiti Putra Malaysia, Serdang 43400, Selangor, Malaysia; ax1895@gmail.com (A.X.L.); ctzue.agb@gmail.com (S.Z.A.G.); 3 School of Fundamental Science, Universiti Malaysia Terengganu, Kuala Nerus 21030, Terengganu, Malaysia; maulidiani@umt.edu.my; 4 Atta-ur-Rahman Institute for Natural Product Discovery, Universiti Teknologi MARA, Puncak Alam Campus, Bandar Puncak Alam 42300, Selangor, Malaysia; medianiahmed47@yahoo.fr

**Keywords:** MD2 pineapple waste, ethanol ratios, antioxidants, α-glucosidase inhibitory, metabolomics

## Abstract

Pineapple (*Ananas comosus*) waste is a promising source of metabolites for therapeutics, functional foods, and cosmeceutical applications. This study strives to characterize the complete metabolite profiles of a variety of MD2 pineapple waste extracts. Metabolomics strategies were utilized to identify bioactive metabolites of this variety prepared with different solvent ratios. Each pineapple waste extract was first screened for total phenolic content, 2,2-diphenyl-1-picrylhydrazyl free radical scavenging, nitric oxide scavenging, and α-glucosidase inhibitory activities. The highest TPC was found in all samples of the peel, crown, and core extracted using a 50% ethanol ratio, even though the results were fairly significant than those obtained for other ethanol ratios. Additionally, crown extracted with a 100% ethanol ratio demonstrated the highest potency in DPPH and NO scavenging activity, with IC_50_ values of 296.31 and 338.52 µg/mL, respectively. Peel extracted with 100% ethanol exhibited the highest α-glucosidase inhibitory activity with an IC_50_ value of 92.95 µg/mL. Then, the extracts were analyzed and the data from ^1^H NMR were processed using multivariate data analysis. A partial least squares and correlogram plot suggested that 3-methylglutaric acid, threonine, valine, and α-linolenic acid were the main contributors to the antioxidant activities, whereas epicatechin was responsible for the α-glucosidase inhibitory activity. Relative quantification further supported that 100% crown extract was among the extracts that possessed the most abundant potential metabolites. The present study demonstrated that the crown and peel parts of MD2 pineapple extracted with 100% ethanol are potentially natural sources of antioxidants and α-glucosidase inhibitors, respectively.

## 1. Introduction

**Figure 1:**
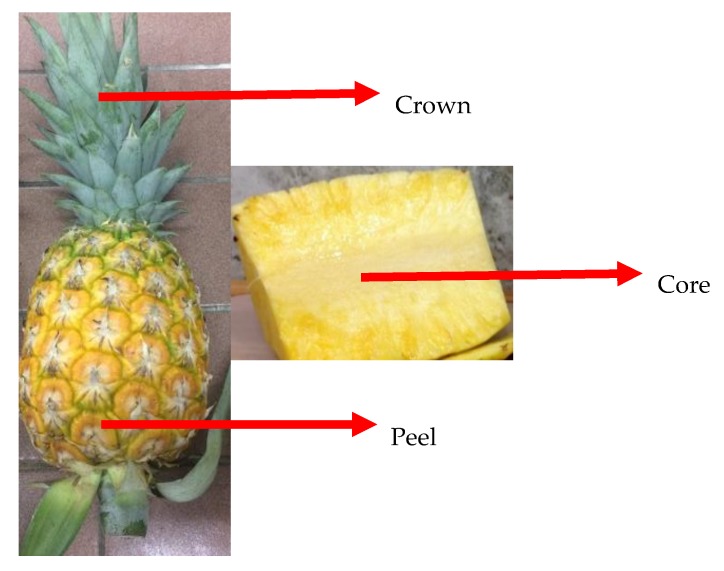
MD2 pineapple.

To date, biowaste products derived from various sources, including fruits have been widely recycled into valuable products ranging from agricultural compost, citric acid production, biofuel, pigment, and bioactive compound production [1]. This recycling method can also effectively resolve an environmental imbalance. An example of such fruit is pineapple (*Ananas comosus*), which belongs to the Bromeliaceae family. Among many other pineapple species planted in Malaysia, the MD2 pineapple is a high-quality hybrid type with an exceptionally sweet taste and flavour and is uniform in size and ripeness [2]. Because of these traits, MD2 is also referred to as “Golden Ripe”, “Super Sweet” and “Gold”. Apart from fresh consumption, the MD2 pineapple has been successfully processed and commercialized as it has own nutritional value including essential mineral composition, vitamins, organic acids and total polyphenol content that offers health benefits [3]. However, the inedible parts of MD2 pineapple waste, including its core, crown, and peel are mostly dumped and then pollute the environment (Figure 1). Recently, research efforts have focused on finding better uses for MD2 pineapple waste as such types of waste generally are rich in phenolic compounds, which are antioxidant agents [4].

Pineapple waste could be a valuable source of important bioactive compounds that have countless beneficial for therapeutic application, including antioxidant, anti-inflammatory and anti-microbial properties [5,6]. These characteristics might be due to the bioactive metabolites such as pigments, sugars, organic acids, amino acids and proteolytic enzyme, bromelain [6]. The ability of pineapple core extracts to scavenge oxidants has been reported in a few studies [7]. Li et al. [4] identified polyphenolics metabolites from pineapple peel (i.e., catechin, epicatechin, gallic acid, and ferulic acid) that contributed to the reduction of oxidative stress-related diseases [4] These metabolites could be integrated into the bioprocess to produce valuable by-products or food ingredients. Despite the growing importance of pineapple waste due to its nutritional, functional and biological properties, information on the primary and especially the secondary metabolites of different parts of MD2 pineapple waste is extremely scarce in the literature, and currently, little is known about the bioactive compounds. The bioactive compounds can have a wide range of variations depending on the environmental conditions, such as the characteristics of the soil, level of humidity, rain intensity, water stress, temperature, fertilizers, and sunlight fluctuation. As a result of variation, the metabolome and the bioactivity of each part of the plant also will be affected [8]. However, despite the importance of studying the MD2 pineapple, little information is available in the literature about its metabolite composition. Furthermore, few studies have addressed the identification of crucial metabolites closely associated with the bioactivities of different parts of the MD2 pineapple waste.

The present study attempts to obtain a comprehensive idea of the key metabolite composition present in waste from different parts of MD2 pineapples. A nuclear magnetic resonance (NMR)-based metabolomics approach will allow us to identify the metabolome, which is important for the studied activities. Recently, metabolomics approach has provided insight into the physiological status of an organism and in the food science field, has been used to assess the quality, authenticity, and safety of food products [9,10]. However, to date, there is no study on the application of a ^1^H NMR-based metabolomics approach to analyze the metabolites in MD2 pineapple waste. Additionally, as the efficiency of metabolite extracted depends on the solvent polarity, we focused on the effects of the solvent ratio on the optimal metabolome extraction and bioactivities of MD2 pineapple.

In this study, MD2 pineapple core, peel, and crown extracted with different solvent ratios (100% ethanol, 70% ethanol and 50% ethanol) were screened for total phenolic content (TPC), 2,2-diphenyl-1-picrylhydrazyl (DPPH) free radical scavenging, NO scavenging and α-glucosidase inhibitory activities. Subsequently, ^1^H NMR-based metabolomics approach was utilized to acquire comprehensive metabolite profiling of the MD2 pineapple core, peel, and crown extracts. To the best of our knowledge, this study provides the first detailed metabolites composition of different parts of MD2 pineapples waste via ^1^H NMR-based metabolomics approach.

## 2. Materials and Methods

### 2.1. Chemical Reagents

Absolute ethanol, analytical grade methanol, dimethyl sulfoxide (DMSO), sodium carbonate, sodium nitroprusside, phosphate buffer saline, sulphanilamide, naphthylethylene diamine dihydrochloride, 85% phosphoric acid, monosodium phosphate, disodium phosphate, glycine, alpha-glucosidase enzyme, deuterated methanol-d4 (CD_3_OD), nondeuterated potassium dihydrogen phosphate (KH_2_PO_4_), deuterium oxide (D_2_O), trimethylsilyl propionic acid-d4 sodium salt (TSP) were supplied by Merck (Darmstadt, Germany) whereas gallic acid, Folin–Ciocalteau reagent, quercetin, 2,2-diphenyl-1-picrylhydrazyl (DPPH), p-nitrophenyl-α-D-glucopyranose (PNPG) were supplied by Sigma (Aldrich, Germany). Deionized water was purified by ultrapure water system from Sartorius (Sartorius AG, Göttingen, Germany).

### 2.2. Plant Material 

A total of 36 pieces MD2 pineapples from 36 different pineapple trees were purchased from a pineapple plantation located at Ayer Hitam, Johor (latitude 1 55′ 00′′ to North and 103 11′ 00′′ to East. In the study area, Johor has a tropical climate which the environment has little seasonal variation, is characterized with wet (high rainfall, annually around 1778 mm and humidity, average relative humidity around 83%) and pretty warm (high temperatures, average daytime temperature 31 °C) [11]. For MD2 pineapple plantations, peat soils were used for planting and drains were built to allow water to flow as this soil easily traps water [11]. The NPK (nitrogen phosphorus potassium) fertilizer was used in the form of granular, applied in a row alongside the plant at every two months after planting, at the rate of 200 kg/ha/year of N, 50 kg/ha/year of P, and 200 kg/ha/year of K [11]. Pineapple fruits were harvested based on their maturity index and skin color, center of fruit and fruit filling (Index 6, with 75% ripe, the appearance transition from light green yellow to golden yellow orange) on July 2018 when it was the ‘middle seasons’ started [12]. The total soluble solids (8.6 to 18.0° Brix) and titratable acidity (0.5 to 0.78%) for the MD2 pineapples were conducted according to the previously reported information [13]. The pineapples were divided into 3 batches with each batch containing 12 pineapples. Each MD2 pineapples were cut and separated to obtain their peel, core, and crown. Each part of the pineapple waste was then cut into smaller pieces and subjected to forced air convection oven drying at 40 °C until completely dried. After drying, they were grinded into fine powder using food processor grinder (HR2056/00, Philips, Malaysia) and kept in the ultra-low temperature freezer (Haier, Middlesex, UK) at −80 °C prior to extraction.

### 2.3. Extraction Method

The ground (5 g) pineapple peel, core and crown were extracted with 100 mL of various ethanol ratios (100%, 50% and 0%) using ultrasound sonication (Thermo-10D ultrasonic cleaner, Fisher scientific, Pittsburgh, PA, USA) at below 40 °C for 1 h. The mixture was then filtered using Whatman No.1 filter paper with 125 mm diameter. After filtration, another 50 mL of solvent was added to the residues for second time sonication under the same condition to maximize the yield. The first and second filtrates were then pooled and concentrated using a rotary evaporator (Hei-Vap, Heidolph, Schwabach, Germany) and lyophilized to remove excess water using freeze dryer (55-4 system Scanvac CoolSafe, Astech, Ireland). All of extracts were stored in the freezer at −80 °C until further analysis.

### 2.4. Total Phenolic Content (TPC) Assay

The TPC of pineapple peel, crown and core extracts was examined using Folin–Ciocalteu method in a 96-well microplate according to the procedure previously reported with some modifications [11]. A volume of 20 µL of sample (100%, 50% and 0% ethanol extracts at 5000 µg/mL concentration) and 100 µL of Folin–Ciocalteu reagent were added into each well and incubated for 5 min. Then, 80 µL of 0.75% sodium carbonate was added to each well and incubated for 20 min in the dark. The absorbance was then measured at 765 nm using Tecan Infinite F200 Pro plate reader (Tecan Group Ltd., Männedorf, Switzerland). This analysis was performed in triplicate. Gallic acid was used as a standard. The same procedure was repeated by replacing the samples with gallic acid at serial dilution with concentration of 0, 0.625, 1.25, 2.5, 5, and 10 µg/mL in the well to plot a standard curve. A standard curve was calibrated with gallic acid and the results were expressed in mg GAE/g crude extract.

### 2.5. 2,2-Diphenyl-1-picrylhydrazyl (DPPH) Radical Scavenging Activity Assay

The antioxidant potential of the pineapple peel, crown and core extracts was determined based on DPPH free radical scavenging assay as previously described [14]. Before the analysis, 3000 µg/mL of stock sample was prepared. The assay was conducted in a 96-well microplate with 50 µL of the test samples prepared in 7 serial dilutions starting with 1000 µg/mL in the well. An aliquot of 100 µL of 0.15 mM DPPH was then added into the well. For the blank sample, 100 µL of methanol was added to obtain accurate sample absorbance. The plate was then incubated for 30 min before measuring the absorbance at 517 nm against the reagent blank by Tecan Infinite F200 Pro plate reader (Tecan Group Ltd., Männedorf, Switzerland). The analysis was performed in triplicate. The same procedure was applied for quercetin which acts as a positive control. The scavenging capacity of the test sample was calculated as the percentage of DPPH inhibition (%) = [(Ao − As)/Ao] × 100 where Ao indicated the absorbance of the reagent blank and As indicated the absorbance of the test samples. The results were expressed in IC_50_ value (µg/mL), which denotes the concentration of sample required to scavenge 50% DPPH free radicals.

### 2.6. Nitric oxide (NO) Radical Scavenging Activity Assay

Another approach of examining the antioxidant capacity of the pineapple peel, crown and core extracts was determined by NO radical scavenging activity assay described previously with slight modifications [15]. Before the analysis, 3000 µg/mL of stock sample was prepared. The assay was also conducted in a 96-well microplate with 60 µL of the test samples prepared in 7 serial dilutions starting with 1000 µg/mL in the well. Then, a volume of 60 µL of 10 mM sodium nitroprusside in phosphate buffer saline was added into the well. For the blank sample, a volume of 60 µL of the phosphate buffer saline was added. The plate was then incubated under light for 180 min. After incubation, 60 µL of freshly prepared Griess reagent (combination of 1% sulphanilamide and 0.1% naphthylethylene diamine dihydrochloride in 2.5% phosphoric acid) was added into each well. The absorbance was then measured at 550 nm by Tecan Infinite F200 Pro plate reader (Tecan Group Ltd., Männedorf, Switzerland). The analysis was performed in triplicate. Gallic acid was employed as the positive control. The percentage of nitrite radical scavenging activity was calculated using the following formula: Percentage of nitric oxide inhibition (%) = [(Ao − As)/Ao] × 100 where Ao indicated the absorbance of the reagent blank and As indicated the absorbance of the test samples. The results were also expressed IC_50_ value (µg/mL), which denotes the concentration of sample required to scavenge 50% nitric oxide free radicals.

### 2.7. α-Glucosidase Inhibition Assay

The α-glucosidase inhibition assay was performed in 96-well microplate as previously described [16]. First, both 30 mM and 50 mM phosphate buffer were prepared at pH 6.5. Then, ρ-nitrophenyl-β-D-glucopyranosidase (PNPG) as a substrate was dissolved in 50 mM phosphate buffer mimics the intestinal fluid. A concentration of 2 M glycine which act as the stopping agent of the reaction was prepared at pH 10. The stock sample extract was prepared at 3000 µg/mL and 7 times serial dilution was performed with starting concentration of 1000 µg/mL in the well. For this assay, an aliquot of 10 µL of sample extract was added into the well, followed by addition of 130 µL of 30 mM phosphate buffer and 10 µL of enzyme. Meanwhile, the extract sample was substituted by DMSO solvent for the negative control. For the blank sample, a volume of 140 µL of 30 mM buffer and 10 µL sample were loaded into the well whereas 140 µL of 30 mM buffer and 10 µL DMSO solvent were loaded for blank solvent. Positive control was added with 130 µL of 30 mM phosphate buffer, 10 µL of quercetin and 10 µL enzyme. The plate was then incubated for 5 min. After incubation period, each well of sample, negative and positive controls were added with 50 µL of PNPG while the rest was added with 50 µL of 30 mM buffer and further incubated for another 15 min. The reaction mixtures were stopped by adding 50 µL of 2 M glycine at pH 10. The absorbance was measured at 405 nm using a Tecan Infinite F200 Pro plate reader (Tecan Group Ltd., Männedorf, Switzerland). The α-glucosidase inhibition activity was expressed as percentage of α-glucosidase inhibition (%) = [(A_n_ − A_s_)/A_n_] × 100% where A_n_ is the difference in absorbance of the negative control and all the blanks while As indicated the absorbance of the test sample.

### 2.8. ^1^H NMR Analysis

The ^1^H NMR were implemented following reported method using 500 MHz Varian INOVA NMR spectrometer (Varian Inc, Palo Alto, CA, USA) functioning at temperature of 26 °C with frequency of 499.887 MHz [16]. Each ^1^H NMR spectrum acquired a width of 20 ppm, which consisted of 64 scans with a 3.53 min acquisition time. For each sample, a relaxation delay of 1.0 s was recorded. The preparation procedure was done by weighing 10 mg of crude extract and placed in a 1 mL Eppendorf tube. Then, 375 µL of CD_3_OD and the same volume of KH_2_PO_4_ buffer in D_2_O (pH 6.0) containing 0.1% TSP were added. The TSP was used as internal reference, TSP = 0.0 ppm. The mixture was then vortexed for 1 min followed by 10 min of ultra-sonication. Next, the mixture was centrifuged at 130× *g* for 10 min to obtain a clear supernatant. A volume of 600 µL of the supernatant was transferred to NMR tube and subjected to ^1^H NMR analysis. All the NMR tubes were labeled and subjected to ^1^H NMR measurements using a predetermined setting for all the samples.

### 2.9. The Bucketing of ^1^H-NMR Spectra and Multivariate Statistical Analysis

Phasing and baseline corrections were performed manually for all the spectra using software built in Varian INOVA NMR spectrometer (Varian Inc., Palo Alto, CA, USA). The bucketing and binning of all the ^1^H NMR spectra were performed automatically into ASCII files by Chenomx software (version 6.2, Edmonton, AB, Canada). The spectra were bucketed at δ 0.04 covering the range from δ 0.50–10.00. The regions of δ 4.70–4.90 and δ 3.23–3.36, which corresponding to the residual signals of water and methanol respectively, were excluded from the analysis.

### 2.10. Statistical Analysis

The experimental results were expressed in mean ± standard deviation of four biological replicates. Analysis of variance (ANOVA) was used to evaluate the significant difference in the results. Pearson correlation test was evaluated to show the relationship between assays. All the statistical analysis was performed using Minitab software (Version 16, Minitab Inc, State College, PA, USA). Principal component analysis (PCA) and partial least square analysis (PLS) were performed by SIMCA-P+ software (version 14.1, Umetrics AB, Umea, Sweden) using Pareto scaling method. The correlogram plot was done using RStudio version 1.0.13 (RStudio, Boston, MA, USA). The size of correlation of tentatively identified metabolites with the bioactivities was determined using the following rules: r values between −0.3 to 0.3 means negligible correlation; from 0.3 to 0.5 (or −0.3 to −0.5) represent low correlation; r values from 0.5 to 0.7 (or −0.5 to −0.7) indicate a moderate correlation; r values from 0.7 to 0.9 (or −0.7 to −0.9) mean a strong or high correlation and, finally, r values between 0.9 and 1.0 (or −0.9 to −1.0) imply a very strong or highest correlation [17]. The changes in metabolite levels were quantitatively evaluated and displayed in a box plot using MetaboAnalyst 3.0 (http://www.metaboanalyst.ca). Tukey’s significant difference multiple comparison test was applied to evaluate the significant differences between the extracts.

## 3. Results and Discussion

### 3.1. TPC of MD2 Pineapple Peel, Crown and Core Extracts

The TPC content of MD2 pineapple peel, crown and core subjected to different ethanol ratio extractions is shown in Table 1, ranging from 3.53 to 12.71 mg GAE/g crude extract. The results demonstrated that crown extracts exhibited the highest TPC recorded at 12.71 mg GAE/g crude extract, followed by peel extracts with 10.73 mg GAE/g crude extract, which both extracts were prepared from 50% ethanol ratio. Core extracts derived from 50% ethanol ratio exhibited the lowest TPC with 4.80 mg GAE/g crude extract.

Abiotic stress, such as temperature and light intensity could also lead to different concentrations of secondary metabolites in plant parts [18]. A study by Nur Asniyati et al. [19] reported that leaves of MD2 pineapple grown in their natural environment showed significantly higher TPC (0.433 mg GAE/g of extract) than those grown in vitro (0.296 mg GAE/g of extract). This is because the light intensity in their natural environment (30,000 to 100,000 lux) was much higher than that in the in vitro environment (approximately 1000 lux). This could explain why the crown extracts exhibited the highest TPC in the current study, their structure and position at the top most part of the pineapple fruit, allows them to receive a greater amount of light than the peel and core. Since photosynthesis takes place in the leaves, the most abundant phenolic biosynthetic pathway precursors can be found in the crown extracts, which has also been well as confirming the accumulation of phenolic compound [20].

In terms of different ethanol ratio extractions, it was observed that the 50% ethanol contributed to the highest TPC in all the three parts of MD2 pineapple waste with significant difference (*p* < 0.05). The result of the current study is in agreement with that of Wang et al. [21], in which they reported that a 50% ethanol ratio extraction under ultrasound sonication treatment for 30 min contribute to the highest TPC from apple pomace. Water and ethanol mixtures are commonly used to extract phenolic compounds due to their ability to dissolve a wide range of compounds, as reported in numerous studies [22,23]. However, the solubility of the phenolic compounds in the solvent is not the only predominant factor in the extraction of TPC. This could explain why a 100% ethanol ratio is not the best extraction solvent for phenolic, rather, a 50% to 80% ratios yields the best extraction of targeted compounds [24]. This is compatible with the results of the current study, in which a 100% ethanol ratio extraction yielded lower TPC than the 50% ethanol out of all the crude extracts.

### 3.2. DPPH Free Radical Scavenging Activity of MD2 Pineapple Peel, Crown and Core Extracts

The results of DPPH free radical scavenging activity of MD2 pineapple peel, crown and core extracts subjected to different ethanol ratio extractions is presented in Table 1. The percentage of inhibition of MD2 pineapple peel, crown and core extracts were varied from 30.13% to 75.57%. Detectable IC_50_ values ranged from 296.31 µg/mL to 386.70 µg/mL, with a quercetin standard of 11.25 µg/mL. Similar to the trend in TPC, the plant parts that showed the most active DPPH free radical scavenging activity were the crown extracted with 100% ethanol ratio (75.57%), followed by the peel (72.67%) and core (49.14%) extracted with 50% ethanol ratio. This could be explained by the fact that phenolic compounds are the major contributors to the antioxidant activity of plant extracts. The chemical structure and spatial conformation of the phenolic compounds, which determine the availability of the hydroxyl group to react with the free radical, is important in evaluating their antioxidant potential [25].

In terms of ethanol ratio extraction, the crown extracted with a 100% ethanol ratio possessed the highest DPPH scavenging activity and resulted in the lowest IC_50_ value (296.31 µg/mL) compared to peel and core extracts. It was found that crown extracted with 0% ethanol showed significantly lower percentage of DPPH inhibition (46.56%) compared to the crown extracted from 50% and 100% ethanol ratio extracts, with no IC_50_ determined. Based on the results, it revealed that low antioxidant activity of 0% ethanol was due to low solubility of the metabolites in water compared to ethanol. The results are interrelated with the TPC content examined in the previous section. It was observed that crown extracted with a 50% ethanol ratio (highest in TPC) did not correspond directly to the highest DPPH free radical scavenging activity in this study. This is because no significant difference (*p* < 0.05) was observed for crown extracted with different ethanol ratios in TPC, suggesting that extracted phenolic compounds varied in property and nature, even though the amounts of yield were relatively equal. Thus, they acted differently in scavenging of DPPH free radical scavenging.

It was also noted that 0% ethanol of all types MD2 pineapple waste extracts were significantly lowest in the DPPH free radicals’ scavenging activity. Similar results were observed in the study by Sun et al. [26], who reported that the 75% ethanol extracted propolis illustrated stronger antioxidant activities than water extract [26]. However, it is also important to note that not only concentration, but also the synergistic effect stemming from the interactions between extracted phenolic compounds directly affected the extract’s antioxidant capacity [27]. The results of the current findings vary compared to those of the few previous studies. Rathnakumar et al. [28] reported that the IC_50_ values of pineapple core and peel were 38.65 µg/mL and 738.3 µg/mL, respectively [29]. The maturation stages of the pineapple could also result in differences in antioxidant activity [13].

### 3.3. Nitric Oxide (NO) Free Radical Scavenging Activity of MD2 Pineapple Peel, Crown and Core Extracts

Another bioassay to test the antioxidant potential of MD2 pineapple peel, crown and core subjected to different ethanol ratio extractions was examined through the NO free radical scavenging activity, the results of which are shown in Table 1. Based on the results, the percentage of NO inhibitory activity of MD2 pineapple peel, crown and core extracted with three different ethanol ratios varied from 27.82 to 70.21%, with a significant difference (*p* < 0.05). The highest percentage of NO scavenging activity was seen in 100% ethanol ratio of crown extracts (70.21%), followed by 0% ethanol ratio of peel (63.60%) and 50% ethanol ratio of core (31.62%) extracts. The detectable IC_50_ ranged from 328.77 to 848.87 µg/mL, with that of gallic acid standard recorded at 3.5 µg/mL.

Generally, the percentage of NO scavenging in all the parts examined was lower compared to the DPPH percentage. This is because the mode of action and scavenging capacity of antioxidants from these plant extracts toward DPPH and NO free radicals are different [29]. Regarding the different ethanol ratio extractions, crown extracted with 100% ethanol contributed to the highest percentage of NO scavenging activity (70.21%) with IC_50_ value of 338.52 µg/mL (Table 1), demonstrating a trend similar to that of DPPH inhibition activity. All core extracts were significantly (*p* < 0.05) lowest in their NO scavenging activity, with no IC_50_ determined.

### 3.4. α-Glucosidase Inhibitory Activity of MD2 Pineapple Peel, Crown and Core Extracts

The α-glucosidase inhibitory activity of MD2 pineapple peel, crown and core extracted with three different ethanol ratios is shown in Table 1. Based on the results, the percentage of α-glucosidase inhibitory activity of MD2 pineapple peel, crown and core extracted with different ethanol ratios ranged from 23.59% to 73.86%. The detectable IC_50_ values ranged from 92.95 µg/mL to 878.75 µg/mL, with a quercetin standard of 0.99 µg/mL (Figure S1). The 100% ethanol ratio peel extract significantly (*p* < 0.05) possessed the highest percentage of α-glucosidase inhibitory activity compared to the 50% and 0% ethanol ratio extracts. It possessed the lowest IC_50_ value (92.95 µg/mL), indicating the strongest capability of inhibiting the action of the α-glucosidase enzyme (Table 1).

From these findings, the 100% ethanol was the most capable solvent of extracting the phytochemical constituents responsible for the α-glucosidase inhibition of MD2 pineapple peel. The results were consistent with those of previous studies in which 100% ethanol extract was found to be the most efficient in α-glucosidase inhibitory activity of *Cosmos caudatus* leaves [30]. While the 50% ethanol ratio extract of crown showed relatively good activities in TPC, DPPH and NO free radical scavenging, it was weak in α-glucosidase inhibitory activity with only 23.59% inhibition capability, which is the lowest among all the plant parts and different ethanol ratio extractions. It is worth noting that although the 50% ethanol ratio extracts of peel, crown and core extracts showed the highest TPC, as discussed earlier, they were weak in α-glucosidase inhibitory activity with inhibition percentages of 44.51%, 23.59% and 46.16%, respectively. This may be due to the fact that the major contributors to α-glucosidase inhibitory activity may not be phenolic compounds, but rather non-phenolic compounds [31]. Further identification of the compounds is required to support this finding. Regardless of the difference in ethanol ratio extractions, all core extracts were found to be significantly (*p* < 0.05) weak in their α-glucosidase inhibitory activity, with no IC_50_ determined.

### 3.5. Visual Inspection of The ^1^H NMR Spectra and Metabolite Identification of MD2 Pineapple Peel, Crown and Core Extracts

**Figure 2:**
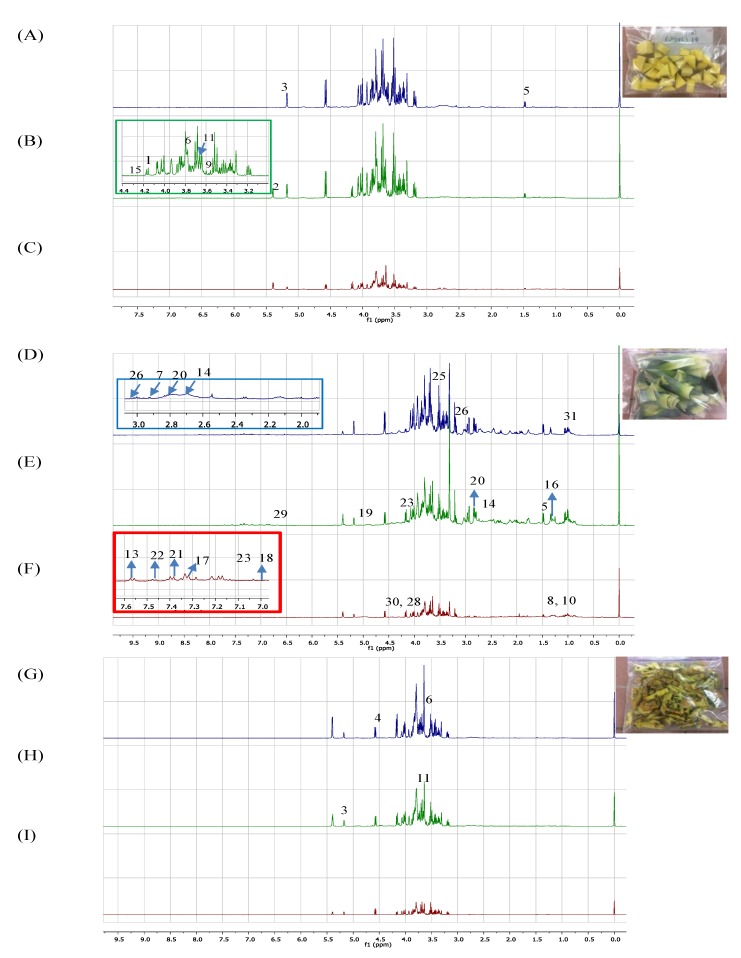
500MHz representative 1H NMR spectra of MD2 pineapple peel, crown and core extracted with 0, 50 and 100% ethanol ratio. (A) 0% ethanol peel; (B) 50% ethanol peel; (C) 100% ethanol peel; (D) 0% ethanol crown; (E) 50% ethanol crown; (F) 100% ethanol crown; (G) 0% ethanol core; (H) 50% ethanol core and (I) 100% ethanol core. See Table 1 for the assignments of the identified metabolites signals.

A detailed analysis based on ^1^H NMR was performed to further study the variation of metabolites and biological activities in MD2 pineapple peel, crown and core extracted using different ethanol ratios. Based on previous studies on pineapple, numerous primary and secondary metabolites were reported primarily on the fruit and leaves, including sugars [32], amino acids [33], fatty acids [34] and phenolic compounds [35,36]. There is limited published data on the metabolites in pineapple crown, peel, and core based on the ^1^H NMR metabolomics approach in particular, and no studies have been published regarding the MD2 pineapple. The representative ^1^H NMR spectra of MD2 pineapple peel, crown and core extracted with 0%, 50% and 100% ethanol ratios are shown in Figure 2. A total of 31 metabolites were identified with their corresponding chemical shifts presented in Table 2.

In the current study, metabolites were identified based on a comparison of chemical shifts assigned in ^1^H NMR spectra with several publicly accessible metabolomics databases, such as the Madison Metabolomics Consortium Database (MMCD; http://mmcd.nmrfam.wisc.edu/), the Human Metabolome Database (HMDB; http://www.hmdb.ca/) peak fitting routine of Chenomx database (v. 8.1, Edmonton, AB, Canada) and previous literature data [16,31,36,37]. Based on visual inspection of the representative ^1^H NMR spectra, different classes of metabolites including sugars, amino acids, organic acids and fatty acids were found to be present. The variation of signal intensity among different parts of MD2 pineapple waste extracted with different ethanol ratios was obvious, especially in the carbohydrate regions. The most intense signals were located in the carbohydrate regions, with chemical shift ranging from δ 3.0 to δ 5.50. This region was dominated by sugars such as fructose, δ sucrose (δ 5.38), α-D-glucose (δ 5.18) and β-D-glucose (δ 4.58). Sukruansuwan and Napathorn [38] demonstrated an abundance of sugars present in pineapple peel and core that are beneficial to fermentation.

The amino acids identified correspond to alanine (δ 1.47), arginine (δ 3.78), asparagine (δ 2.93), threonine (δ 1.34), glycine (δ 3.57), valine (δ 1.06), isoleucine (δ 3.66), phenylalanine (δ 7.30) and tryptophan (δ 7.54). Most of the amino acids identified fall into the regions between δ 1.0 and 3.6, except phenylalanine (δ 7.30) and tryptophan (δ 7.54) (aromatic amino acids). Meanwhile, two organic acids and a lipid, known as malic acid (δ 4.30), citric acid (δ 2.72) and α-linolenic acid (δ 1.26) were also identified, respectively. In addition, signals in aromatic regions were the least intense observed in the ^1^H NMR spectra for all parts of MD2 pineapple waste regardless of the ethanol ratio used. Phenolic compounds in pineapple waste extracts were also tentatively identified and corresponded to the aromatic regions in the crude extract based on their 1D NMR and in previous *A. comosus* [L.] Merr. studies [39]; they were ferulic acid (δ 7.34), gallic acid (δ 7.04), epicatechin (δ 6.12), protocatechuic acid (δ 7.40 and δ 6.90), benzoic acid (δ 7.70 and δ 7.46), syringic acid (7 δ.37), phenylacetic acid (7.42 m) and fumaric acid (6.50). The doublets at δ 7.52 and δ 7.02 may be attributed to vanilic acid (protons H-2 and H-6). Referring to Table 2, it is interesting to note that regardless of the ethanol ratio used in the extraction, the MD2 pineapple crown showed variation compared to peel and core as demonstrated by the presence of amino acid (asparagine, δ 2.93) and phenolics (catechin, δ 2.82). Otherwise, citric acid was not identified in MD2 pineapple core.

### 3.6. Metabolite Variations in MD2 Pineapple Peel, Crown and Core Extracts

**Figure 3:**
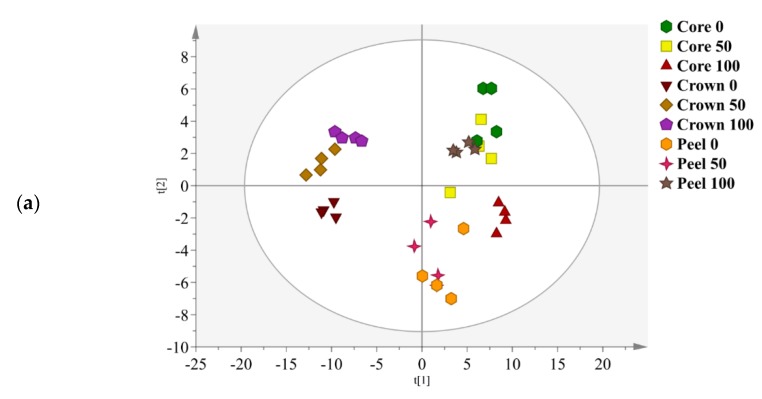

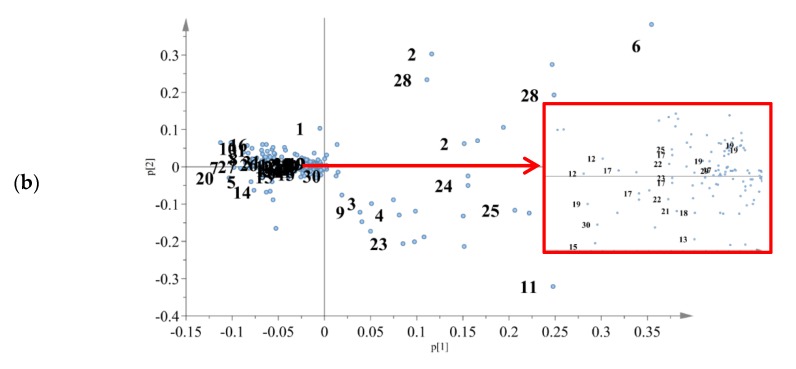
(**a**) The PCA score plot for MD2 pineapple peel, crown and core extracted with 0, 50 and 100% ethanol ratio. (**b**) The PCA loading plot. See Table 1 for the assignments of metabolites.

In the current study, ^1^H NMR data were subjected to principal component analysis (PCA) to discriminate metabolite variations of different parts of MD2 pineapple waste extracted with different ethanol ratios. PCA provides the primary evaluation of the relationships between the samples using the unsupervised clustering method [14]. The separation of samples into clusters can be examined in the PCA score plot whereas metabolites that contributed to the separation are revealed in the PCA loading plot. Both the PCA score and loading plots of MD2 pineapple peel, crown and core extracted with different ethanol ratios are illustrated in Figure 3. The PCA model showed good fitness and high predictability with an R^2^X of 0.99 and a Q^2^ of 0.96, respectively. The total variance of the model was 82.7%, for which PC1 contributed 68.2% and PC2 contributed 14.5%.

Based on the score plot, MD2 pineapple crown extracts were well clustered and clearly separated from peel and core extracts by PC1 (Figure 3a). Meanwhile, crown extracted with 50% and 100% ethanol were clearly distinguishable from crown extracted with a 0% ethanol by PC2. Peel extracted with a 100% ethanol was well clustered and separated from the 50% and 0% ethanol ratio peel extracts by PC2. Among the core extracts, the 100% ethanol showed good cluster, distinguishing them from the 50% and 0% ethanol ratios of core extracts by PC2. Compared to the 100% ethanol extracts, core and peel extracts from 50% and 0% were more dispersed and less clustered.

According to the PCA loading plot, the majority of the metabolites were loaded at the left quadrant which corresponds to higher metabolites constituents in MD2 pineapple crown extracts (Figure 3b). The metabolites that resulted from separation of crown extracts from peel and core extracts by PC1 including sucrose (2), glyceric acid (28), syringic acid (24), phenylacetic acid (25), α-D-glucose (3), glycine (9), β-D-glucose (4) and isoleucine (11). In the upper left quadrant, the metabolites are more concentrated which are attributed by 50% and 100% crown extracts. Other metabolites, including fructose (1) and α-linolenic acid (16), were located in the upper left quadrant of the PC2 loading plot also contributed to the separation of crown extracts.

### 3.7. Classification of MD2 Pineapple Peel, Crown and Core Extracts by Partial Least Squares Analysis (PLS)

**Figure 4:**
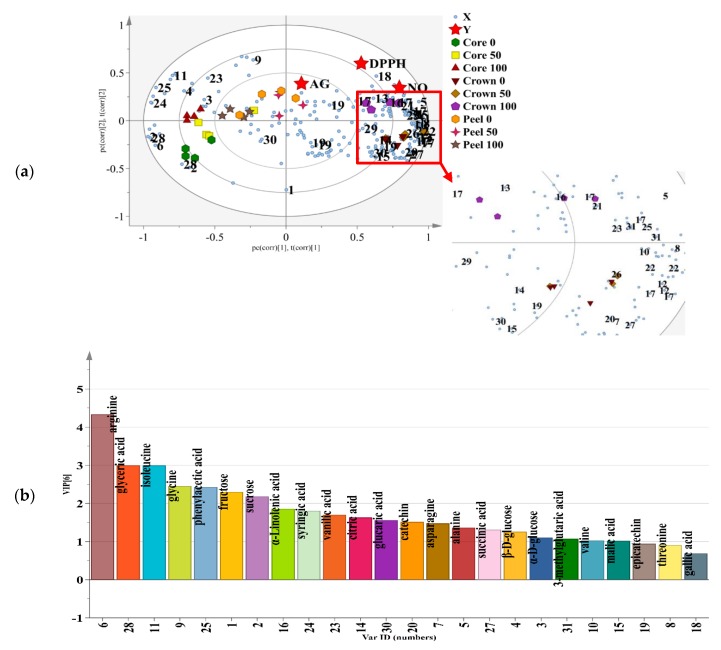
(**a**) The PLS biplot describing the correlation of MD2 pineapple peel, crown and core extracted with 0, 50 and 100% ethanol ratio and biological activities. See Table 1 for the assignments of metabolites. (**b**) Metabolites with variable importance in the projection (VIP) value ≥ 0.7 identified from MD2 pineapple peel, crown and core extracted with 0, 50 and 100% ethanol ratio.

PLS analysis as a supervised approach was applied to further investigate the association between metabolites and biological activities in MD2 pineapple peel, crown and core extracted with different ethanol ratios. A PLS biplot with a combination of score and loading plots was constructed to illustrate the distinction between samples and variables contributing to the separations [31]. The chemical shifts of metabolites from the ^1^H NMR dataset were represented by X variables, and the biological activities (DPPH, NO and AG) were represented by Y variables. As shown in Figure 4a, the PLS biplot showed good fitness (R2Y = 0.95) and moderate predictive ability (Q2 = 0.67).

The DPPH and NO free radical scavenging activities (Y variables) were located at the positive side of the plot and were also projected close to 100% ethanol ratio-extracted crown suggesting that they were strongly correlated. This is consistent with results in bioassay studies where 100% ethanol ratio extracted crown was the most active in both DPPH and NO scavenging activities. Although crown extracted with a 50% ethanol ratio was projected nearest to the NO variable, there was no significant difference found in the bioassay with 100% ethanol ratio extracted crown. The α-glucosidase inhibitory variable is positioned nearest to the peel extracted with the 100% ethanol ratio, suggesting their strong correlation. Cores extracted with different ethanol ratios were clustered on the negative sides of the plot and furthest apart from the Y variables, indicating weak correlation with the biological activities.

Based on the PLS biplot, the metabolites that contributed the most to the higher DPPH and NO free radical scavenging activities were 3-methylglutaric acid (31), catechin (20)**,** gallic acid (18)**,** threonine (8), valine (10), fructose (1), and α-linolenic acid (16) (compounds with a VIP value ≥ 0.7, Figure 4b) which were found in the 100% ethanol ratio crown extracts. Catechin (20) and gallic acid (18) are suggested as the phenolic compound responsible for the DPPH and NO free radical scavenging activities due to its closest projection to the Y variables. Furthermore, it can be observed that ferulic acid (17), tryptophan (13) and protocatechuic acid (21) also had positive correlations with DPPH and NO free radical scavenging. However, they have VIP values less than 0.7 which might indicate that they are compounds that have a lower contribution to the bioactivities. The lower chamber of the positive side of PC1 was occupied by the 50% and 0% ethanol ratio crown extracts. The metabolites that had a positive correlation with the DPPH and NO activities were asparagine (7), citric acid (14), succinic acid (27) and epicatechin (19). Malonic acid (26), benzoic acid (22) and phenylalanine (12) were still considered to contribute comparably for the DPPH and NO activities of the 50% and 0% ethanol ratio crown extract.

In addition, epicatechin which was positioned closed to the clusters of peel extracts is believed to be one of the metabolites contributing to the α-glucosidase inhibitory activity, (regardless of the ethanol ratio used). Previous studies have shown that flavonoid acts as a good α-glucosidase inhibitor due to the saturation of hydroxyl groups at ring B; and hydroxyl substitution at position at 3′and 5′ of ring A and C [40]. Epicatechin was also reported to inhibit the α-glucosidase enzyme [36]. Glycine, sucrose, α-D-glucose, β-D-glucose, glucaric acid, vanillic acid, phenylacetic acid, syringic acid, glyceric acid, arginine, and isoleucine (VIP value ≥ 0.7, Figure 4b) were well correlated with the peel and core extracts (regardless of the ethanol ratio used) but were located on the negative side of PC1 and they were negatively correlated with the observed Y variables. These compounds are worth analyzing as they might indirectly affect the bioactivities. Metabolites including glycine (9), α-D-glucose (3) β-D-glucose (4), vanillic acid (23), phenylacetic acid (25), syringic acid (24), isoleucine (11) located on the positive side of PC2 are believed to have some indirect positive effects on improving the tested bioactivities, unlike the metabolites located on the negative side of PC2.

The PLS model was validated using 200 permutation tests to evaluate its goodness of fit and predictive power. The permutation test results of the DPPH scavenging activity (Supplementary, Figure S2a) showed that Y-intercepts R^2^ and Q^2^ were 0.342 and −0.715; while those of the NO scavenging activity (Supplementary, Figure S2b) were 0.338 and −0.694, respectively, and those of the α-glucosidase inhibitory activity (Supplementary, Figure S2c), the Y-intercepts of R^2^ and Q^2^ were 0.243 and −0.627. These results indicated that the constructed PLS model was valid and not over fitting, exhibiting good predictive abilities. Based on Figure S3a–c, the relationships between the observed versus the predicted plots of the Y variable, DPPH, NO scavenging activity and α-glucosidase inhibitory activity showed regression lines with R^2^ values of 0.81, 0.78 and 0.85, respectively. The DPPH scavenging activity showed a relatively low root mean square error of estimation (RMSEE) value of 8.06687 and a root mean square of error of cross-validation (RMSEcv) value of 8.3657; the NO scavenging activity demonstrated an RMSEE value of 7.89406 and RMSEcv value of 7.89853 and the α-glucosidase inhibition activity showed an RMSEE value of 6.71054 and an RMSEcv value of 10.6637 (Table 3). The current results show that these models are good and can be applied to predict outcomes of future experiments [41].

### 3.8. Relationship Between Antioxidant and α-Glucosidase Inhibitory Activities with Bioactive Metabolites

**Figure 5:**
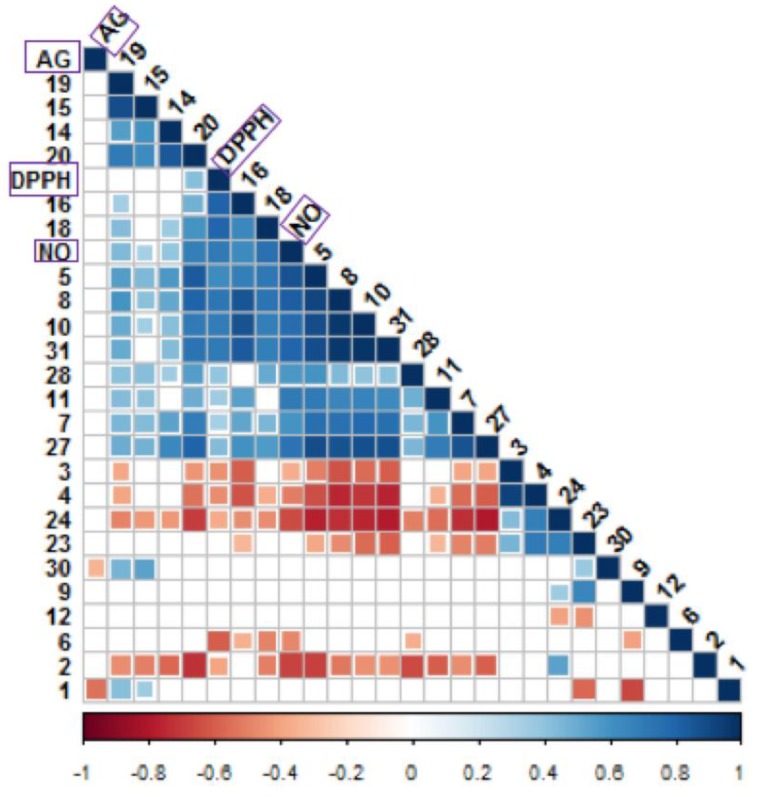
Correlogram visualizing correlation between metabolites analyzed using ^1^H NMR and biological activities. Correlation with *p*-value > 0.05 are considered insignificant and are represented by the blank white space. Color and size of the squares are proportional to the correlation coefficients. Positive correlations are shown in blue (different shades; dark blue with the strongest correlation) whereas negative correlations in red (ranging from light red to red; dark red with the weakest correlation). Assignment of metabolites: 1, fructose; 2, sucrose; 3, α-D-glucose; 4, β-D-glucose;6, arginine; 5, alanine; 7, asparagine; 8, threonine; 9, glycine; 10, valine; 11, isoleucine; 12, phenylalanine; 13, tryptophan; 14, citric acid; 15, malic acid; 14, citric acid; 15, malic acid; 16, α-linolenic acid; 17, ferulic acid; 18, gallic acid; 19, epicatechin; 20, catechin; 23, vanillic acid; 24, syringic acid; 27, succinic acid; 28, glyceric acid; 31, 3-methylglucaric acid.

The correlation was further scrutinized by computing the Pearson correlation coefficient (*p* < 0.05) and a correlogram was constructed (Figure 5) to verify the strength of the relationship among several variables [17]. However, the analysis was narrowed down by emphasizing metabolites with VIP values ≥ 0.7; hence, a total of 24 metabolites was selected (Figure 4b). The obtained correlogram showed strong and significant correlations among alanine (5), threonine (8), valine (10), gallic acid (18) and 3-methylglutaric acid (31) and two biological activities (DPPH and NO). Glyceric acid (28), asparagine (7), catechin (20), succinic acid (27) and isoleucine (11) were mildly correlated with DPPH but strongly correlated with NO. In contrast, epicatechin (19), malic acid (15) and citric acid (14) were mildly correlated with the NO but has no correlation observed with DPPH. α-Linolenic acid (16) was significantly correlated with both DPPH and NO scavenging but not with α-glucosidase inhibitory activity. This compound possessed good correlation with DPPH scavenging activity, suggesting it is as a good antioxidant. Previous study found that wheat leaves with an abundance of α-linolenic acid exhibit considerable antioxidant activity [42]. Fructose (1) and glucaric acid (30) were weakly linked with α-glucosidase inhibitory activity. All of these metabolites displayed good VIP values; hence, it can be suggested that they contribute significantly to the observed bioactivities.

### 3.9. Quantification of Metabolites in MD2 Pineapple Peel, Crown and Core Extracts

**Figure 6:**
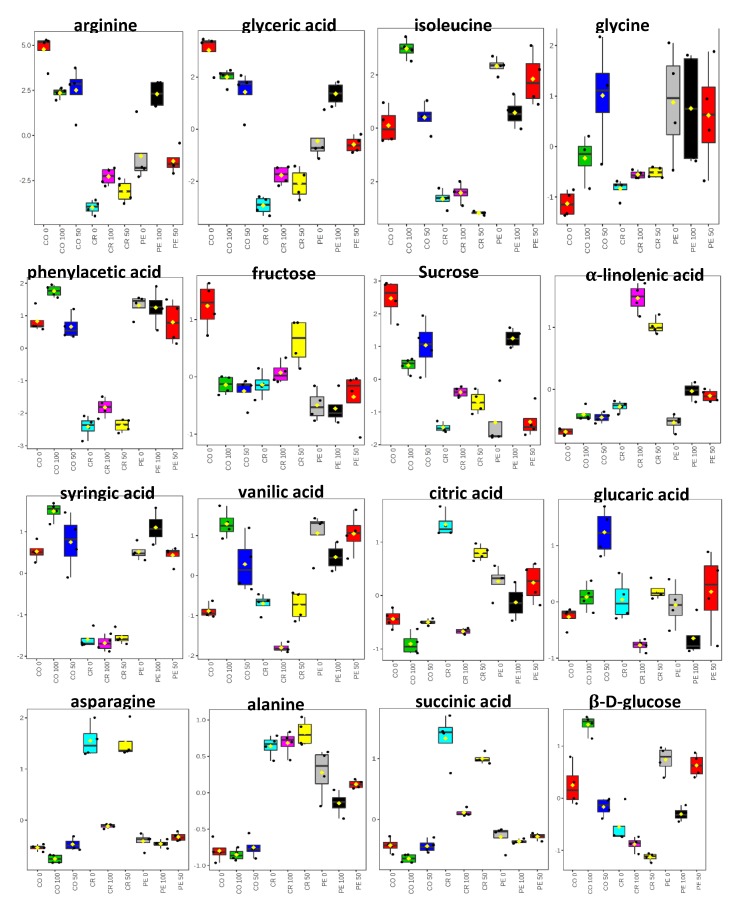

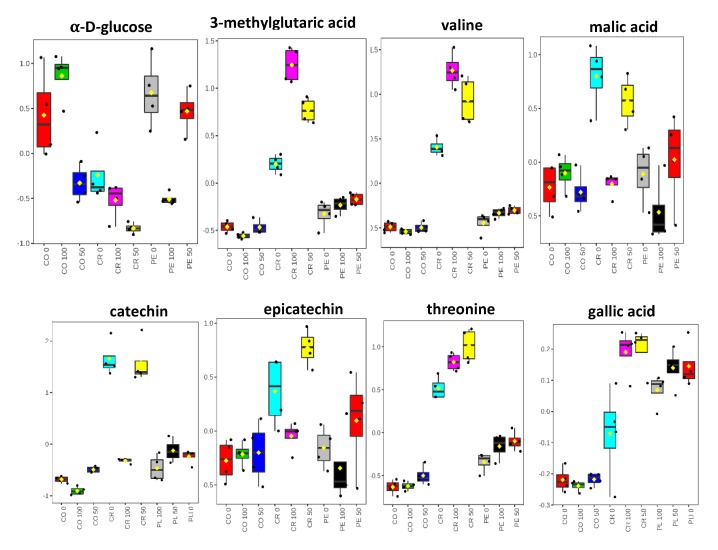
Boxplots of tentatively identified metabolites showing their relative quantification associated with MD2 pineapple waste extracts using 1H NMR spectra binned data. The metabolites are those with variable importance in the projection (VIP) value >0.7 of MD2 pineapple extracted with 0, 50 and 100% ethanol ratio Waste extracts are prepared from CO = core; CR = crown and PL = peel.

Relative quantification was performed through normalization to the internal reference (TSP) and depicted in box plots (Figure 6). The mean peak area calculated from ^1^H NMR was used to determine the intensity of those metabolites exhibiting PLS VIPs ≥ 0.7 [31]. This discussion focuses on crown extracts from the 100% ethanol ratios that showed the highest potency in DPPH and NO scavenging activity and on peel extracted with 100% ethanol that exhibited the highest α-glucosidase inhibitory activity. The high levels of the carboxylic acids and essential amino acids may be linked to the quantities of 3-methylglutaric acid, threonine and valine in the crown extracts of 100% ethanol ratio. The α-linolenic acid was found in significant amount in the crown extracts which was reported earlier to possess antioxidant and anti-inflammatory properties [43].

By relating the quantification data with the PLS biplot (Figure 4) and correlogram plot (Figure 5), these variation in metabolites 3-methylglutaric acid (31), α-linolenic acid (16), valine (10), and threonine (8) in crown extracts from a 100% ethanol ratio may provide an explanation for the higher biological activities of DPPH and NO free radical scavenging. Epicatechin (19) even identified in low concentration in peel extracted with 100% ethanol is believed to be one of the marker metabolites contributing to α-glucosidase inhibitory activity as shown in the PLS biplot, while fructose (1) and glucaric acid (30) are considered to have a weak contribution to α-glucosidase inhibitory activity.

## 4. Conclusions

In conclusion, the current study demonstrated that MD2 pineapple peel, crown, and core extracted with 0%, 50%, and 100% ethanol ratios possessed variations in TPC, DPPH free radical scavenging activity, NO free radical scavenging activity, and α-glucosidase inhibitory activity. Based on the biological assays, MD2 pineapple crown extracted with a 100% ethanol ratio presented better scavenging effects on DPPH and NO free radical, scavenging, suggesting that it is a better antioxidant compared to peel and core. However, peel extracted with a 100% ethanol ratio exhibited better α-glucosidase inhibition properties with an IC_50_ value less than 100 µg/mL, which may act as α-glucosidase inhibitor. Regardless of different ethanol ratio extractions, MD2 pineapple core presented the lowest activity in all the bioassays examined. The ^1^H NMR metabolomics successfully investigated the metabolite profiles of the MD2 pineapple peel, crown, and core extracted from different ethanol ratios. The current study provides valuable information regarding sample preparation methods for waste portions and the potential use of metabolomics for studying the correlation between plant metabolites and bioactivity. However, a more comprehensive study is needed to further understand the underlying mechanism of bioactive compounds obtained from MD2 pineapple waste extracts, and their toxicity and bioavailability after ingestion in order to help develop their applications as a functional food.

## Figures and Tables

**Table 1:** The bioactivities of pineapple peel, crown and core extracted with 0, 50 and 100% ethanol.

Part	Ethanol	TPC	DPPH Scavenging Activity		Nitric Oxide Scavenging Activity		α-Glucosidase Inhibition	
	Ratio (%)	(mg GAE/g crude extract)	% inhibition	IC50(µg/mL)	% inhibition	IC50(µg/mL)	% inhibition	IC50 (µg/mL)
Peel	0	10.62 ± 0.37 ^a^_b_	68.96 ± 1.07 ^b^_a_	386.70 ± 17.55 ^a^	63.60 ± 0.85 ^a^_a_	658.19 ± 16.06 _b_	54.99 ± 2.88 ^b^_b_	878.75 ± 96.19 ^a^_a_
	50	10.73 ± 0.14 ^a^_b_	72.67 ± 1.51 ^a^_a_	339.23 ± 14.61 ^b^_a_	46.74 ± 1.56 ^b^_b_	ND	44.51 ± 3.35 ^c^_a_	ND
	100	7.97 ± 0.63 ^b^_b_	71.24 ± 1.38 ^ab^_b_	353.10 ± 21.34 ^ab^_a_	44.43 ± 0.75 ^c^_b_	ND	73.86 ± 5.39 ^a^_a_	92.95 ± 6.09 ^b^_b_
Crown	0	12.15 ± 0.62 ^a^_a_	46.56 ± 1.51 ^c^_b_	ND	51.16 ± 1.01 ^b^_b_	848.87 ± 34.18 ^a^_a_	65.24 ± 1.47 ^a^_a_	552.44 ± 68.55 ^a^_b_
	50	12.71 ± 1.15 ^a^_a_	72.19 ± 1.91 ^b^_a_	341.44 ± 32.26 ^a^_a_	65.86 ± 7.38 ^a^_a_	328.77 ± 30.17 ^b^	23.59 ± 1.67 ^b^_b_	ND
	100	12.22 ± 0.83 ^a^_a_	75.57 ± 0.81 ^a^_a_	296.31 ± 12.74 ^b^_b_	70.21 ± 1.72 ^a^_a_	338.52 ± 25.32 ^b^	63.44 ± 4.38 ^a^_b_	651.49 ± 57.35 ^a^_a_
Core	0	3.53 ± 0.12 ^c^_c_	30.13 ± 2.00 ^c^_c_	ND	27.82 ± 1.87 ^b^_c_	ND	32.26 ± 3.01 ^c^_c_	ND
	50	4.80 ± 0.23 ^a^_c_	49.14 ± 0.61 ^a^_b_	ND	31.62 ± 1.69 ^a^_c_	ND	46.16 ± 1.88 ^a^_a_	ND
	100	4.15 ± 0.20 ^b^_c_	35.00 ± 1.32 ^b^_c_	ND	31.31 ± 1.73 ^a^_c_	ND	41.10 ± 1.49 ^b^_c_	ND
Standard				11.24 ± 0.39 (Quercetin)		3.51 ± 0.51 (Gallic Acid)		0.99 ± 0.14 (Quercetin)

The values represent means ± standard deviation based on four biological replicates. The superscript is to compare the same part of plant with different ethanol ratio; the subscript is to compare different parts of the plant with same ethanol ratio. Mean with different superscript and subscript letters are significantly difference (*p* < 0.05); ND is not determined.

**Table 2:** Tentatively identified metabolites of ^1^H NMR spectra from MD2 pineapple peel, crown and core extracted with 0, 50 and 100% ethanol ratio.

Metabolites	Chemical Shift (δ)	Parts of MD2 Pineapple Waste/Ethanol Ratio (%)								
		Peel			Crown			Core		
		0	50	100	0	50	100	0	50	100
*Sugar*										
(1) Fructose	4.17(d)	+	+	+	+	+	+	+	+	+
(2) Sucrose	5.38(d), 4.02(t)	+	+	+	+	+	+	+	+	+
(3) α-D-glucose	5.18(d)	+	+	+	+	+	+	+	+	+
(4) β-D-glucose	4.58(d)	+	+	+	+	+	+	+	+	+
*Amino Acids*										
(5) Alanine	1.47(d)	+	+	+	+	+	+	+	+	+
(6) Arginine	3.78(t)	+	+	+	+	+	+	+	+	+
(7) Asparagine	2.93(d)	-	-	-	+	+	+	-	-	-
(8) Threonine	1.34(d)	+	+	+	+	+	+	+	+	+
(9) Glycine	3.57(s)	+	+	+	+	+	+	+	+	+
(10) Valine	1.06(d)	+	+	+	+	+	+	+	+	+
(11) Isoleucine	3.66(d)	+	+	+	+	+	+	+	+	+
(12) Phenyalanine	7.30(d), 7.36(d)	+	+	+	+	+	+	+	+	+
(13) Tryptophan	7.54(d)	+	+	+	+	+	+	+	+	+
*Organic acids*										
(14) Citric acid	2.72(d)	+	+	+	+	+	+	-	-	-
(15) Malic Acid	4.30(dd)	+	+	+	+	+	+	+	+	+
*Lipids*										
(16) α-linolenic acid	1.26(s)	+	+	+	+	+	+	+	+	+
*Phenolic compounds*										
(17) Ferulic acid	7.34(d), 7.27(s), 7.10(dd), 6.93(d), 6.39(d)	+	+	+	+	+	+	+	+	+
(18) Gallic acid	7.04(s)	+	+	+	+	+	+	+	+	+
(19) Epicatechin	6.12(d), 6.09(d), 5.00(s), 4.34(m)	+	+	+	+	+	+	+	+	+
(20) Catechin	2.82(dd)	-	-	-	+	+	+	-	-	-
(21) Protocathechuic acid	7.40 (d) 6.90 (d)	-	-	-	+	+	+	-	-	-
(22) Benzoic acid	7.70(t) 7.46 (dd)	-	-	-	+	+	+	-	-	-
(23) Vanillic acid	7.52 (d) 7.02(d) 3.90 (s)	-	-	-	+	+	+	-	-	-
(24) Syringic acid	7.37(s) 3.98 (s)	+	+	+	+	+	+	+	+	+
(25) Phenylacetic acid	7.42 (m) 7.30 (m) 3.50 (s)	+	+	+	+	+	+	+	+	+
(26) Malonic acid	3.10(s)	+	+	+	+	+	+	-	-	-
(27) Succinic acid	2.42(s)	+	+	+	+	+	+	+	+	+
(28) Glyceric acid	4.14(m) 3.82(m)	+	+	+	+	+	+	+	+	+
(29) Fumaric acid	6.50(s)	-	-	-	+	+	+	-	-	-
(30) Glucaric acid	4.22(d) 4.10(d) 3.92 (t)	+	+	+	+	+	+	+	+	+
(31) 3-methylglutaric acid	2.18(m) 0.90(d)	-	-	-	+	+	+	-	-	-

+: presence; -: absence; d: double; dd: doublet of doublet, m: multiplet, s: singlet; t: triplet.

**Table 3:** PLS model validation.

Biological Assays	No. of Components	R2Y	Q2Y	R2Y Intercepts	Q2Y Intercepts	RMSEE	RMSEcv
DPPH	2	0.817	0.668	0.342	−0.715	8.06687	8.3657
Nitric oxide scavenging	2	0.817	0.668	0.338	−0.694	7.89406	7.89853
α-glucoside	2	0.817	0.668	0.243	−0.627	6.71054	10.6637

## Supplementary Materials

The following are available online at https://www.mdpi.com/2304-8158/9/2/173/s1, Figure S1: (a) Extraction yield; (b) Total phenolic content; (c) DPPH free radical scavenging; (d) NO free radical scavenging; (e) α-Glucosidase inhibitory activities of MD2 pineapple peel, crown and core extracted with 0, 50 and 100% ethanol, Figure S2: Validation of PLS model using permutation test (100 permutations) of DPPH, NO and α-Glucosidase inhibitory activities, Figure S3: PLS derived relationship between observed vs. predicted of DPPH, NO, α-Glucosidase inhibitory activities.
