# Supplementary material for: Potentially Bioactive Metabolites from Pineapple Waste Extracts and Their Antioxidant and α-Glucosidase Inhibitory Activities by 1H NMR
Source: Foods. 2020 Feb 11;9(2):173. doi: 10.3390/foods9020173 (PMC7073707; doi:10.3390/foods9020173)
Supplement: Supplementary file 1 [file foods-09-00173-s001.pdf]

Article

Potentially Bioactive Metabolites from Pineapple Waste Extracts and Their Antioxidant and α-Glucosidase Inhibitory Activities by ^{1}H NMR

Awanis Azizan [1], Lee Ai Xin [2] Nur Ashikin Abdul Hamid [3], M. Maulidiani [4], Siti Zulaikha Abdul Ghaffar [5], Nur Khaleeda Zulaikha Zolkeflee [2] and Faridah Abas [2,*]

1. Laboratory of Natural Products, Institute of Bioscience, Universiti Putra Malaysia, 43400 Serdang, Selangor, Malaysia; faridah_abas@upm.edu.my; awanis_azizan@yahoo.com; khaleeda_zulaikha@yahoo.com; shikinhamid89@yahoo.com
2. Department of Food Science, Faculty of Food Science and Technology, Universiti Putra Malaysia, 43400 Serdang, Selangor, Malaysia; ctzue.agb@gmail.com; ax1895@gmail.com
3. School of Fundamental Science, Universiti Malaysia Terengganu, 21030 Kuala Nerus, Terengganu, Malaysia; maulidiani@upm.edu.my

* Correspondence: faridah_abas@upm.edu.my; Tel.: (+603-97698343)

Received: date; Accepted: date; Published: date

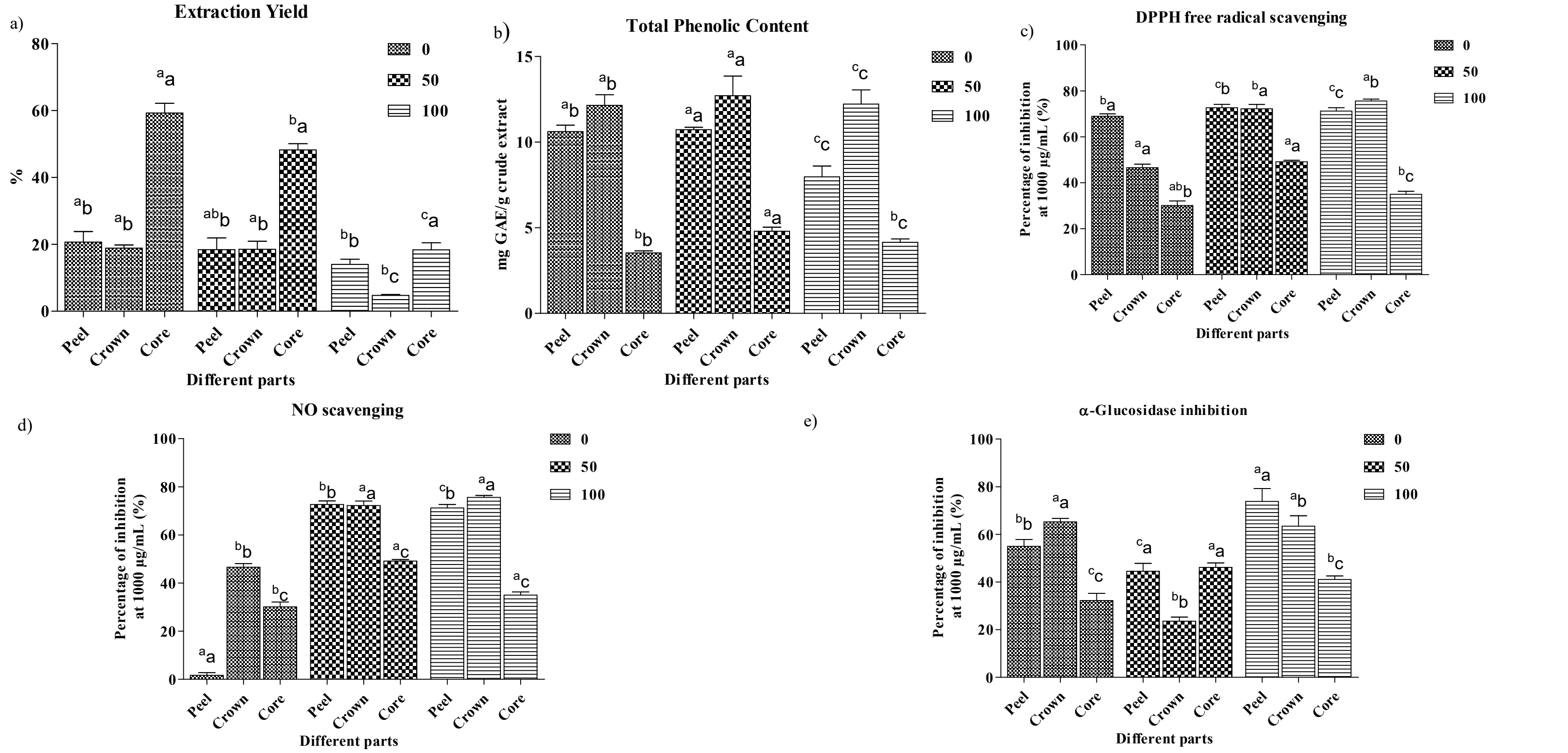

Figure S1. (**a**) Extraction yield; (**b**) Total phenolic content; (**c**) DPPH free radical scavenging; (**d**) NO free radical scavenging; (**e**)α-Glucosidase inhibitory activities of MD2 pineapple peel, crown and core extracted with 0, 50 and 100% ethanol. Values are means ± standard deviation based on four biological replicates. The superscript is to compare the same part of plant with different ethanol ratio extraction; and the subscript is to compare the different parts of the plant with same ethanol ratio extraction. Mean with different superscript and subscript letters are significantly different ($p < 0.05$).

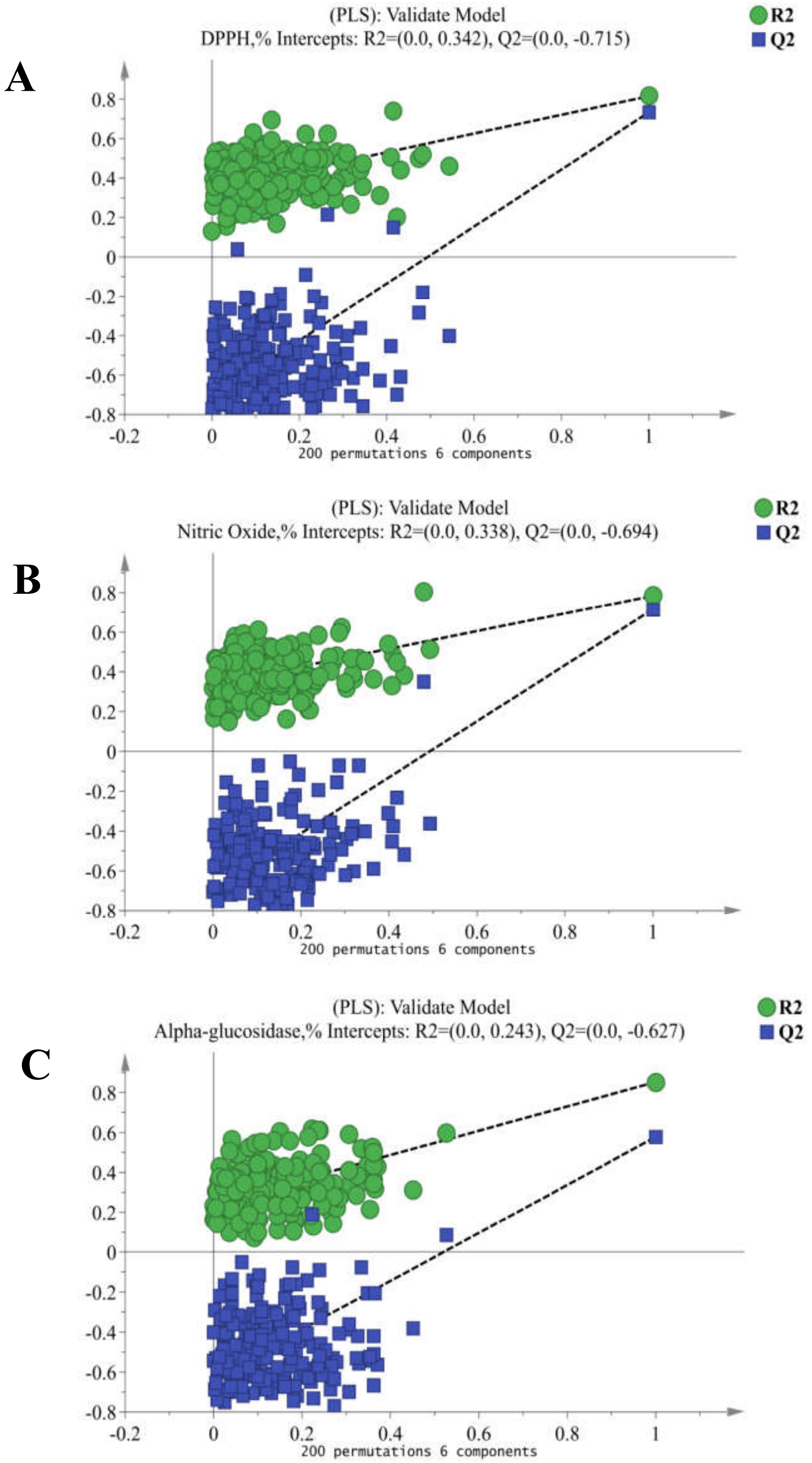

Figure S2. Validation of PLS model using permutation test (100 permutations) of DPPH, NO and α-Glucosidase inhibitory activities.

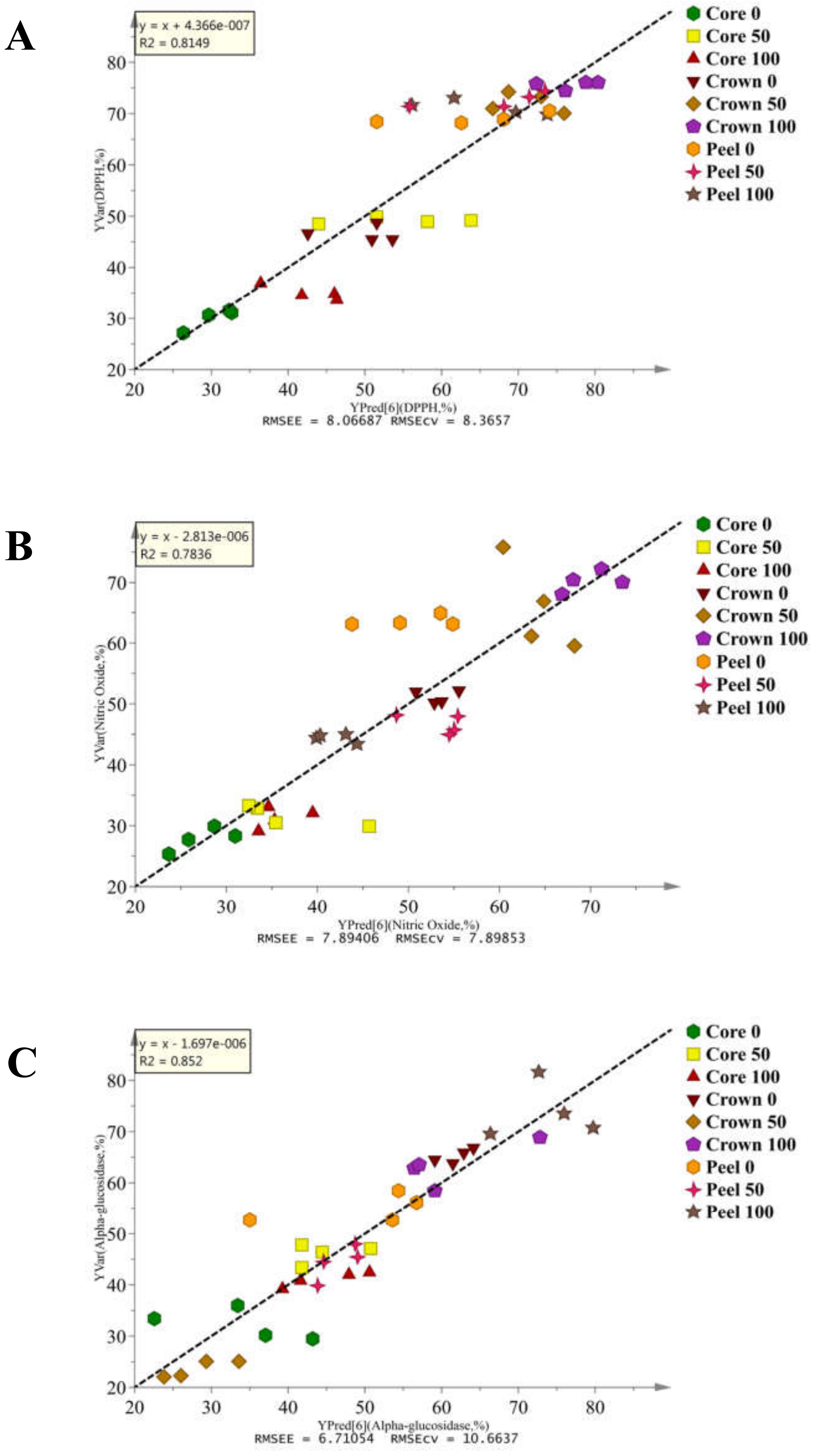

Figure S3. PLS derived relationship between observed vs predicted of DPPH, NO, α-Glucosidase inhibitory activities.
